# Elevated Plasma Soluble ST2 Is Associated with Heart Failure Symptoms and Outcome in Aortic Stenosis

**DOI:** 10.1371/journal.pone.0138940

**Published:** 2015-09-21

**Authors:** Patrizio Lancellotti, Raluca Dulgheru, Julien Magne, Christine Henri, Laurence Servais, Nassim Bouznad, Arnaud Ancion, Christophe Martinez, Laurent Davin, Caroline Le Goff, Alain Nchimi, Luc Piérard, Cécile Oury

**Affiliations:** 1 GIGA Cardiovascular Sciences, Heart Valve Clinic, Department of Cardiology and Radiology, University of Liège Hospital, and University of Liège, Liège, Belgium; 2 Gruppo Villa Maria Care and Research, Lugo (RA), Italy; 3 Department of Cardiology, CHU Dupuytren, and INSERM 1094, Faculté de Médecine de Limoges, Limoges, France; Brigham and Women's Hospital, Harvard Medical School, UNITED STATES

## Abstract

B-type natriuretic peptide (BNP) is often used as a complementary finding in the diagnostic work-up of patients with aortic stenosis (AS). Whether soluble ST2, a new biomarker of cardiac stretch, is associated with symptomatic status and outcome in asymptomatic AS is unknown. sST2 and BNP levels were measured in 86 patients (74±13 years; 59 asymptomatic, 69%) with AS (<1.5 cm^2^) and preserved left ventricular ejection fraction who were followed-up for 26±16 months. Both BNP and sST2 were associated with NYHA class but sST2 (>23 ng/mL, AUC = 0.68, p<0.01) was more accurate to identify asymptomatic patients or those who developed symptoms during follow-up. sST2 was independently related to left atrial index (p<0.0001) and aortic valve area (p = 0.004; model R^2^ = 0.32). A modest correlation was found with BNP (r = 0.4, p<0.01). During follow-up, 29 asymptomatic patients (34%) developed heart failure symptoms. With multivariable analysis, peak aortic jet velocity (HR = 2.7, p = 0.007) and sST2 level (HR = 1.04, p = 0.03) were independent predictors of cardiovascular events. In AS, sST2 levels could provide complementary information regarding symptomatic status, new onset heart failure symptoms and outcome. It might become a promising biomarker in these patients.

## Introduction

Aortic stenosis (AS) is the most common valvular heart disease in western countries. Risk stratification in asymptomatic patients and identifying characteristics of poor prognosis in symptomatic subjects have become key elements in the evaluation of AS [1–3]. Aortic valve replacement (AVR) is indicated in patients with severe AS when symptoms and/or left ventricular (LV) systolic dysfunction (LV ejection fraction < 50%) develop [4–5]. However, LV ejection fraction often remains normal for long despite latent and potentially irreversible alterations in LV myocardial structure and function. Such LV remodelling process is already observed in patients with moderate AS, precedes symptoms development and can trigger biomarkers release (i.e. B-type natriuretic peptide (BNP)) [6]. In AS, the BNP level correlates with valve area, diastolic function, functional status and symptomatic deterioration and may improve risk stratification [7–9]. ST2, a member of the interleukin-1 receptor family, is a novel biomarker of mechanical stress. ST2 is expressed both in a soluble isoform (measured in serum) and in a transmembrane receptor isoform for which IL-33 serves as a ligand [10]. ST2 expression is upregulated in isolated cardiomyocytes exposed to mechanical strain and in explanted aortic valve of patients with non-rheumatic AS [11–12]. Serum soluble ST2 (sST2) levels are elevated and associated with outcomes in patients with acute myocardial infarction and heart failure, independently of BNP [13–19]. Whether sST2 is increased in relation to the symptomatic status and represents an independent marker of poor prognosis in AS is unknown. In the present study, we evaluated sST2 in patients with AS as a potential marker of disease severity, new onset heart failure symptoms and outcome.

## Materials and Methods

The present study consisted of patients with moderate to severe AS (aortic valve area <1.5 cm^2^) and preserved left ventricular (LV) ejection fraction (>50%) examined at our Heart Valve Clinic, CHU of Liège. All enrolled patients agreed to participate in our blood sample collection (Biobank) for valvular heart disease. The institutional review board (« Comité d'Ethique Hospitalo-Facultaire Universitaire de Liège ») approved the protocol and all patients gave written informed consent. Demographics and clinical data were recorded in our Institutional electronic medical record at the time of initial visit in the echo lab. Use of cardiac medications, laboratory results, and symptomatic status at the time of the initial echocardiogram were recorded. Absence of symptoms was confirmed by a normal exercise testing. None of the patients had concomitant moderate or severe valvular conditions other than AS, previous valve surgery, complex congenital heart disease, supravalvular or subvalvular aortic stenosis, hypertrophic cardiomyopathy, plasma creatinine level >13 mg/dL, significant arrhythmia, acute and chronic inflammatory disease.

A comprehensive transthoracic echocardiography was performed using VIVID 7 ultrasound system (General Electric Healthcare, Little Chalfont, UK) in all patients. All Doppler-echocardiographic recordings were stored in digital format on a dedicated workstation for off-line subsequent analysis. All echocardiographic measurements were performed as previously described by our group. Of note, LV stroke volume was calculated using both the Doppler (LV outflow tract area x LV outflow tract velocity—time integral measured by pulsed-wave Doppler) and the volumetric (bi-apical Simpson’s method) methods. Moreover, multiple transducer positions were used to record peak aortic jet velocities. The highest transaortic velocity was used for tracing of the time-velocity integral and to calculate pressure gradients. For each measurement, at least two cardiac cycles were averaged. The peak E-wave of the mitral inflow was measured using pulsed-wave Doppler. Tissue Doppler imaging with pulsed-wave Doppler at the level of septal and lateral mitral annulus was used to measure e’ velocities. The average of septal and lateral mitral annulus e’ peak velocity was used to calculate the E/e’ ratio.

Venous blood samples for BNP and sST2 measurements were drawn before echocardiography, after 10 minutes of supine rest. Chilled ethylenediaminetetraacetic acid tubes were centrifuged immediately at 1000 x g (4°C) for 15 minutes. Separated plasma samples were processed for BNP measurements by immunoassay (Biosite, Beckman Coulter, San Diego, California). The inter- and intra-assay variation was 5% and 4%, respectively. The assay detection limit was 1 pg/ml. A predictive risk score, including BNP, gender and aortic valve peak velocity, was calculated for each patient according to the recent work of Monin et al [9]. The ratio between measured serum BNP level and maximal normal BNP level for age and sex (BNP ratio) was also calculated for each patient [20,21]. Soluble ST2 was measured by a sandwich double monoclonal antibody ELISA method (Quantikine**®** ELISA, R&D Systems, Minneapolis, MN) according to manufacturer’s instructions. The inter- and intra-assay variation was 6% and 5%, respectively.

Follow-up information was obtained from interviews with the patients, their relatives or their physicians every six to twelve months, according to current guidelines [4,5]. The beginning of follow-up was considered the date of the initial echocardiogram at our institution and the end points were the occurrence of new onset heart failure symptoms related events (death, dyspnea, acute pulmonary edema) in the asymptomatic patients.

Data are given as mean values ± standard deviation (SD); categorical variables are described as number and percentages. Differences between groups were analyzed for statistical significance with the 1-way analysis of variance (ANOVA), χ^2^ test, or Fisher exact test as appropriate. Correlations between echocardiographic data and biomarkers were assessed with linear regressions. Independent predictors of biomarker levels were obtained with the use of stepwise multiple linear regressions. Stepwise logistic regression analysis was used to determine the predictors of symptomatic status. Probabilities of end point-free survival were obtained by Kaplan-Meier estimates for the 2 groups and then compared by a 2-sided log-rank test. Cox proportional-hazards models were used both in individual and multivariable analyses to identify the independent predictors of the occurrence of end-points. Variables with a univariable value of p<0.10 were incorporated into the multivariable models. The selection of variables included in the multivariate model was performed with a special care. To avoid colinearity among a subset of several variables measuring the same phenomenon (e.g., aortic valve area, peak aortic pressure gradient, mean aortic pressure gradient), we entered in the multivariate models the variable that had the strongest association with event on univariable analysis. Values of p<0.05 were considered significant. Receiver-operator characteristic (ROC) curve analysis was performed to determine the cut-off values that best distinguished the issue (e.g., symptoms, death or combined events) (AUC: Area under the curve). All statistical analyses were performed with STATISTICA version 10 (StatSoft Inc, Tulsa, Okla).

## Results

**Fig 1:**
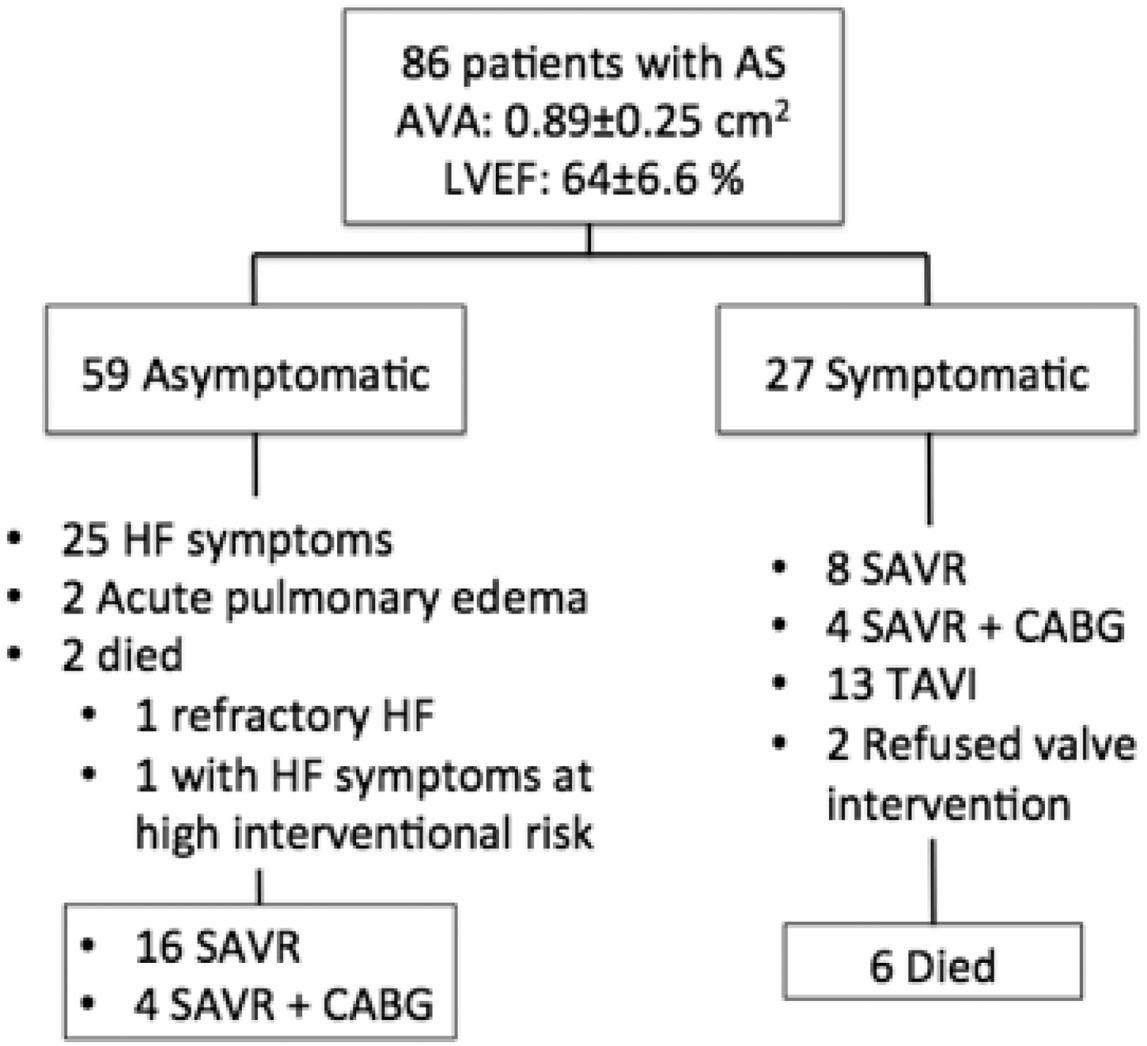
Study population and follow-up.

Of the 86 enrolled patients, 27 (31%) were classified as symptomatic at baseline (i.e. inclusion visit) (Fig 1). Patients were followed-up for 26±16 months. During this period, a total of 8 (9%) patients died from cardiovascular death. Of the 27 symptomatic patients, 12 had surgical aortic valve replacement (4 with revascularization), 13 underwent a transcatheter aortic valve implantation with a CoreValve bioprosthesis (high-surgical risk), and 2 refused any intervention. Six of them died during follow-up and 2 were hospitalized for worsening symptoms (1 in acute atrial fibrillation). Of the 59 asymptomatic patients, 25 patients developed heart failure symptoms (dyspnea in 23, dyspnea and angina in 3), 2 were hospitalized for acute pulmonary edema, 20 underwent aortic valve replacement combined with coronary artery by-pass grafting in 4, and 2 died (1 from refractory heart failure and 1 with heart failure symptoms treated medically because of prohibitive high surgical risk). Hence, a total of 29 patients developed new onset heart failure symptoms.

**Fig 2:**
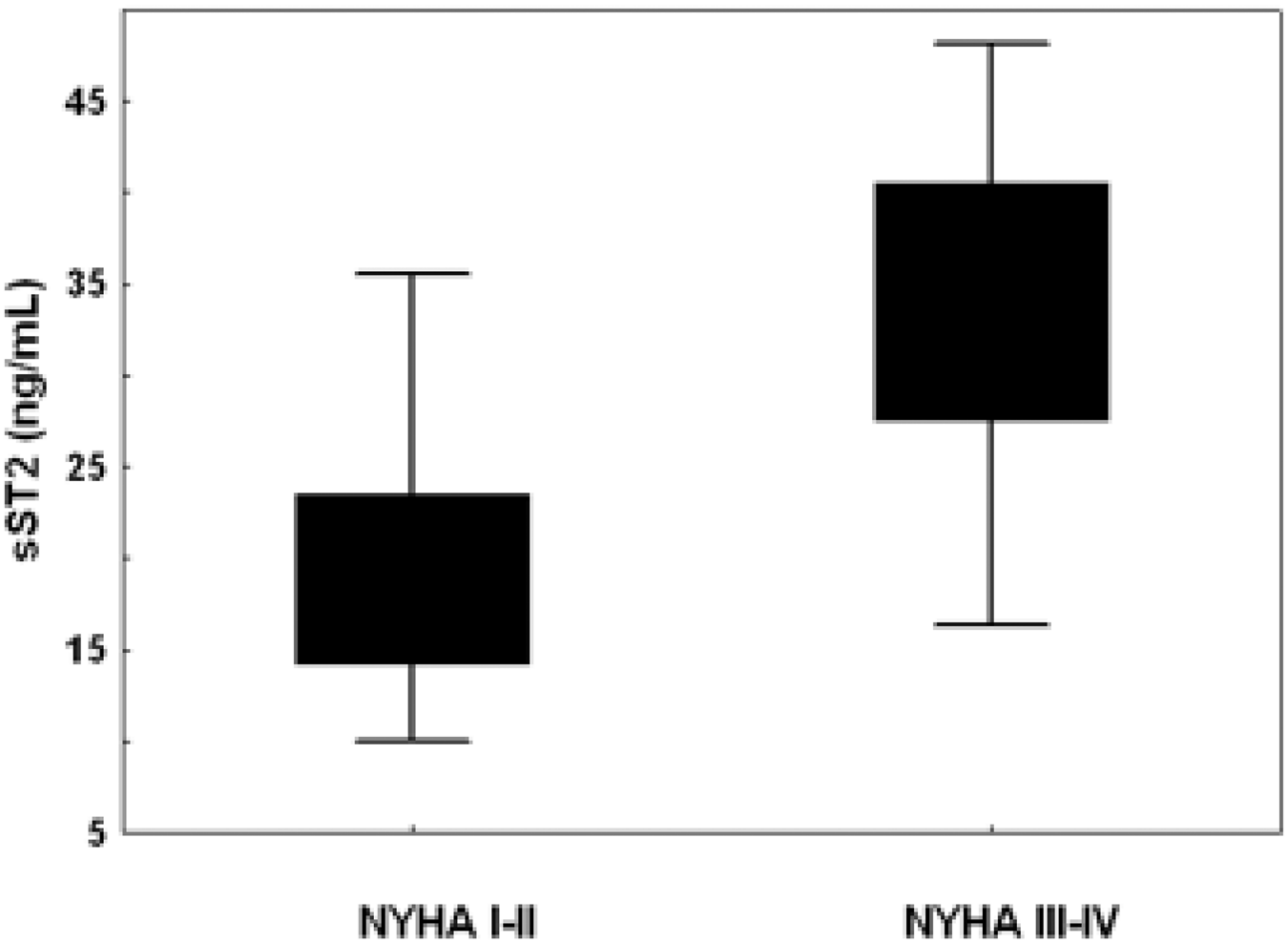
Plasma levels of sST2 at study entry in association with NYHA class.

Compared with the 59 (69%) asymptomatic patients, symptomatic patients at study entry did not differ in gender and presence of risk factors but were older (p = 0.03), had higher aortic pressure gradients (p<0.001), smaller aortic valve area (p<0.001) and higher BNP (p<0.001) and sST2 levels (p = 0.006) (Table 1). BNP and sST2 levels were not statistically different according to gender. BNP was well correlated with age (r = 0.34, p<0.001), which was also true for sST2 though at a lower extent (r = 0.26, p = 0.02). Both biomarkers increased with the symptomatic status (BNP: 100±122 vs. 270±322 pg/mL; sST2: 21±8 vs. 27±11 ng/mL; p<0.001 for all) and the NYHA class (BNP: NYHA class I 100±122, class II 141±125, class III-IV 358±256 pg/mL; sST2: NYHA class I 18±4, class II 20±8, class III-IV 33±9 ng/mL; p<0.001 for class I/II vs. III-IV) (Fig 2). Risk score and BNP ratio were also higher in symptomatic patients (p<0.05). In multivariable logistic regression analysis, after adjustment for age, severity of AS, BNP, left atrial size, E/e’, and LV mass, independent determinants of symptoms were sST2 (odds-ratio [OR] = 1.1, 95% confidence interval [CI] = 1.0–1.3; p = 0.037) and mitral E/e’ (OR = 1.35, CI = 1.02–1.8; p = 0.025). Similar results were obtained when BNP ratio was used instead of age and BNP (sST2: OR 1.07, CI = 1.008–1.15; p = 0.028; E/e’: OR 1.22, CI = 1.07–1.40; p = 0.0036). Using ROC curve analysis, the best cut-off value to identify symptomatic patients was 23 ng/mL for sST2 (AUC = 0.68, sensitivity = 60%, specificity = 74%, p<0.01).

**Table 1:** Demographic, Clinical and Echocardiographic characteristics according to the symptomatic status at study entry and follow-up.

Variables	Asymptomatic all along, (n = 30, 35%)	Symptomatic at follow-up, (n = 29, 34%)	Symptomatic at study entry, (n = 27, 31%)	P value
**Clinical variables**				
Age—years	70±16	72±13	82±6	<0.01
Male gender—no (%)	22 (73)	18 (62)	16 (59)	0.49
Hypertension—no (%)	19 (63)	20 (67)	20 (74)	0.68
Diabetes mellitus—no (%)	4 (13)	6 (21)	8 (30)	0.32
Hypercholesterolemia—no (%)	16 (53)	16 (55)	10 (37)	0.33
Current smoking—no (%)	7 (23)	9 (31)	13 (48)	0.14
**Aortic Stenosis Severity**				
Aortic valve area—cm^2^	1.03±0.2	0.90±0.22	0.73±0.19	<0.01
Peak aortic velocity—m/sec	3.7±0.6	3.9±0.6	4.3±0.6	<0.01
Mean pressure gradient—mm Hg	33±11	42±15	47±15	<0.01
**LV function**				
Indexed LV end-diastolic volume—ml/m^2^	52±18	53±20	55±27	0.97
Indexed LV end-systolic volume—ml/m^2^	21±8	20±8	20±6	0.96
LV ejection fraction—%	65±7	67±5	63±6.5	0.23
LV mass—g/m^2^	80±20	94±22	112±29	<0.01
Mitral E/e’	11±5	12±5	17±6	<0.01
Left atrial area index	9.1±3	11±3	14±5	<0.01
**Biomarkers**				
BNP (pg/mL)	76±74	126±154	270±322	<0.01
BNP ratio	0.97±1.05	1.7±3.3	2.9±3.7	0.04
Risk score	10±1.6	11.5±1.6	12.6±1.7	<0.01
sST2 (ng/mL)	17±4.4	24±10	27±11	<0.01

Values are means ± SD. LV denotes left ventricular.

**Fig 3:**
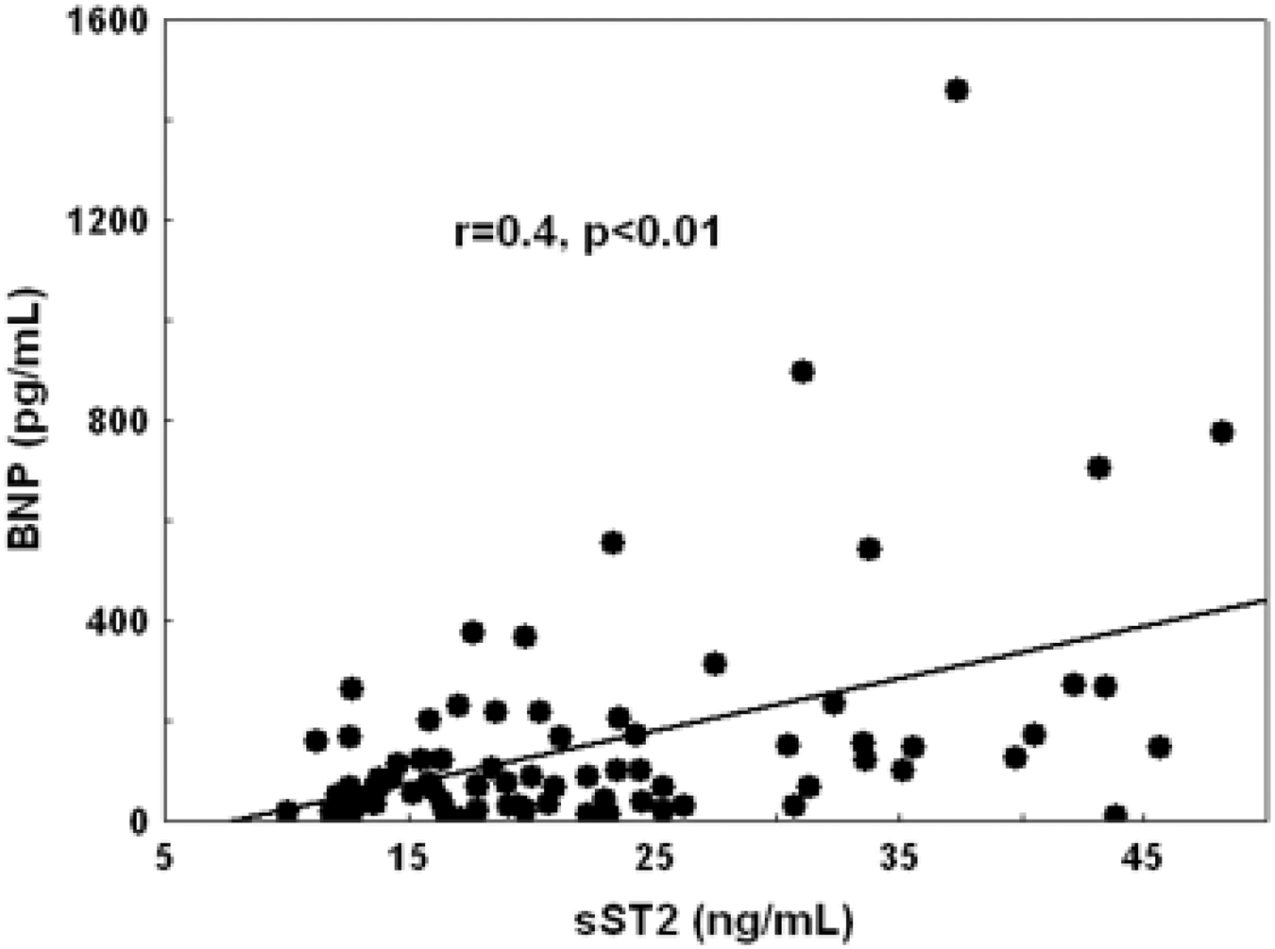
Correlation between sST2 and BNP levels.

In the overall population, plasma levels of BNP and sST2 were correlated with the severity of AS, the degree of LV hypertrophy, the diastolic burden (left atrial area, E/e’) and with the LV ejection fraction (Table 2). sST2 levels were also modestly correlated with BNP levels (r = 0.4, p<0.01) (Fig 3). With multiple linear regression analysis, left atrial index (p<0.0001) and aortic valve area (p = 0.004; model R^2^ = 0.32) were correlated with sST2 levels. Left atrial index (p = 0.025) and E/e’ (p = 0.03; model R^2^ = 0.29) were determinants of BNP. In asymptomatic patients, mean aortic pressure gradient and E/e’ were correlated with BNP, whereas aortic valve area, LV mass, and left atrial size were correlated with sST2. E/e’ (p<0.001; model R^2^ = 0.47) was the only independent determinant of BNP. Aortic valve area (p = 0.006) and LV mass (p = 0.004) were independent determinants of sST2 (model R^2^ = 0.27).

**Table 2:** Echocardiographic correlates of biomarkers.

	Entire population				Asymptomatic patients				Symptomatic patients			
Variables	Log BNP		Log sST2		Log BNP		Log sST2		Log BNP		Log sST2	
	r	p	r	p	r	p	r	p	r	p	r	p
**Aortic Stenosis Severity**												
Aortic valve area	0.36	<0.01	0.33	<0.01	0.23	0.07	0.29	0.029	0.26	0.022	0.21	0.048
Peak aortic velocity	0.27	0.01	0.21	0.06	0.24	0.06	0.28	0.039	0.14	0.28	0.15	0.23
Mean pressure gradient	0.28	0.008	0.28	0.11	0.32	0.012	0.37	0.006	0.11	0.49	0.08	0.59
**LV function**												
LV end-diastolic volume	0.22	0.16	0.08	0.64	0.23	0.17	0.06	0.72	0.17	0.38	0.22	0.09
LV end-systolic volume	0.19	0.25	0.06	0.71	0.19	0.25	0.014	0.93	0.21	0.10	0.19	0.11
LV ejection fraction	0.30	0.01	0.28	0.034	0.14	0.33	0.03	0.83	0.53	0.038	0.52	0.02
LV mass	0.29	0.016	0.43	<0.01	0.04	0.76	0.37	0.01	0.21	0.34	0.27	0.06
Mitral E/e’	0.45	<0.01	0.26	0.036	0.66	<0.001	0.22	0.13	0.14	0.51	0.11	0.59
Left atrial area index	0.49	<0.01	0.49	<0.01	0.24	0.086	0.37	0.008	0.44	0.035	0.41	0.03

LV denotes left ventricular.

In the group of asymptomatic patients, those who experienced events (29 patients) during follow-up tended to be older (p = 0.086), had more severe AS (higher aortic peak jet velocity (p<0.001), smaller aortic valve area (p = 0.001)), larger left atrium (p = 0.011), higher BNP (p = 0.013) and sST2 (p = 0.001) levels (Table 3). For the other clinical and echocardiographic parameters, no significant correlation with the outcome was found (p > 0.1 for all). In the multivariable analysis, peak aortic jet velocity (HR = 2.7, p = 0.007) and sST2 level (HR = 1.04, p = 0.03) were predictive of the outcome. When BNP ratio was used in the multivariable model, peak aortic jet velocity (HR = 2.8, p = 0.007) and sST2 (HR = 1.04; p = 0.043) remained predictive of the outcome. Using the previously identified cut-off (23 ng/mL) in the multivariable model, yielded to similar results where only peak aortic jet velocity (HR = 2.9, CI = 1.47–5.7, p = 0.002) and sST2 > 23 ng/mL (HR = 2.42, CI = 1.07–5.44, p = 0.033) remained independent predictors. When the sST2 tertiles were used in the univariable models, significant differences were obtained between T1 and T3 (p = 0.026) but not between T1 and T2 (p = 0.17) or T2 and T3 (p = 0.09) in terms of outcome.

**Table 3:** Univariable data included in the multivariable outcome models in asymptomatic patients.

Variables	Onset of Heart failure symptoms	
	HR (95 percent CI)	P
Age	1.03 (0.90–1.06)	0.086
Aortic valve area	…	…*
Peak aortic velocity	2.8 (1.6–5.07)	<0.001
Left atrial area index	1.19 (1.04–1.38)	0.011
BNP	2.5 (1.21–5.23)	0.013
sST2	1.05 (1.02–1.09)	0.001

HR denotes Hazard-Ratio, CI denotes confidence interval, LV left ventricular.

*The ellipsis means that the aortic valve area, though significant in our univariable analysis (p = 0.001), was not included in the multivariable model because it was less predictive than peak aortic velocity for heart failure symptoms (p<0.001).

## Discussion

The main findings of this study are: (1) sST2 appears to be a new promising biomarker to separate symptomatic from asymptomatic patients with AS, (2) sST2 is related to AS severity and the extent of diastolic burden as assessed by the left atrial area, (3) high sST2 levels are likely associated with outcomes in asymptomatic AS.

In the present study, there was a good association between the symptomatic status and plasma levels of sST2. Interestingly, sST2 levels gradually increased with the NYHA class. Conversely, although BNP was univariately associated with the NYHA class and correlated modestly with sST2 levels, it did not emerge as an independent determinant of the symptomatic status. Although adjusted for age and sex, BNP ratio [20, 21] did not improve the predictive accuracy of BNP. sST2 could thus be considered as an accurate discriminator between early symptoms of heart failure and normal effort tolerance. Similarly, both BNP (BNP ratio) and sST2 levels univariately predicted the onset of symptoms during follow-up in asymptomatic patients with AS. However, in the multivariable Cox regression analysis, only sST2 predicted symptomatic deterioration over time. Our data extended those described in patients with heart failure in whom sST2 concentrations correlated with clinical indices of severity, such as NYHA functional class [14–16]. BNP is known to be produced by the myocardium in response to pressure overload whereas sST2 protein is released under chronic inflammatory conditions and in response to cardiac mechanical stress [10–12]. sST2 acts as a “decoy receptor” for IL-33, negatively regulating IL-33 signalling at membrane bound ST2. IL-33 bound to sST2 is removed from the biologically active pool and can no longer serve its functions. IL-33 exerts antihypertrophic effects in cultured cardiomyocytes that are antagonized by administration of sST2, and reduces myocardial fibrosis and cardiomyocyte hypertrophy in response to pressure overload in mice [11]. In patients with pressure overload (AS) hypertrophy, diastolic load is the predominant hemodynamic factor that contributes to ST2 production [22]. Main sources of circulating sST2 include the endothelial cells and cardiomyocytes [23]. Moreover, inflammatory conditions of AS may enhance the capacity of these cells to secrete sST2 [24]. In our study, sST2 related to indexes of diastolic burden (left atrial size, E/e’), LV hypertrophy, severity of AS and LV systolic function (ejection fraction); factors that are the hallmark of a more advanced disease process. Similar findings were observed in patients with myocardial infarction, acute dyspnea or heart failure [14–16, 25]. Interestingly, it was recently shown that ST2 expression levels were markedly up-regulated in non-rheumatic AS compared with aortic regurgitant valves, suggesting that IL-33/ST2 pathway may be involved in the pathophysiology of AS [12]. Furthermore, in addition to its role as a decoy receptor for IL-33, excessive amounts of sST2 may induce direct effects, modulating extracellular matrix remodeling and turnover [26], progression of AS and consequent clinical deterioration.

The prognostic power of circulating sST2 has been demonstrated in patients with acute myocardial infarction or in the context of acute dyspnea with or without heart failure [13,15,17,18, 25]. Elevated sST2 levels were predictive of clinical deterioration and short and mid-term prognosis in an additive manner to BNP. In the present study, sST2 levels were also associated with increased cardiovascular risk. In the group of asymptomatic AS patients, sST2 and peak aortic jet velocity predicted symptom-free survival, whereas other clinical or echocardiographic variables or biomarkers (BNP or BNP ratio) did not. Asymptomatic AS patients with a plasma sST2 level >23 ng/mL were more likely to develop symptoms and require aortic valve surgery during follow-up. Interestingly, all these patients were truly asymptomatic since they had a normal exercise test at study entry. These results demonstrate that sST2 could be used to enhance the predictive value of BNP in patients with AS [27,28]. This is probably related to the fact that the IL-33/ST2 pathway and sST2 directly participate in the regulation of extracellular matrix remodelling and inflammation, two major events involved in the pathophysiology of AS.

We acknowledge that our study has some limitations. Despite the examination of a reasonable number of patients with AS, the subgroup of symptomatic AS patients remained relatively small. sST2 measurements were not repeated during the follow-up period, and therefore the prognostic value of serial sST2 changes could not be assessed. The relationship between ST2 and global and regional myocardial function was not assessable. In order to confirm the association of sST2 with AS, further studies that would analyse serial changes of sST2 during patient follow-up are required.
